# SELF-DIRECTED AGEISM OUTCOMES ASSOCIATED WITH AGEISM-REDUCTION INTERVENTIONS: A SCOPING REVIEW

**DOI:** 10.1093/geroni/igad104.2709

**Published:** 2023-12-21

**Authors:** Rachel Scrivano, Shannon Jarrott

**Affiliations:** Boston College, Boston, Massachusetts, United States; The Ohio State University, Columbus, Ohio, United States

## Abstract

Self-directed ageism refers to stereotyping, prejudice, and discrimination toward oneself based on age or perceived age. Self-directed ageism research is limited but growing as the conceptualization of ageism evolves and the implementation of ageism-reduction interventions increases. A scoping review was conducted to explore self-directed ageism outcomes associated with ageism-reduction interventions among populations measured. The PRISMA-ScR checklist guided study methods. A systematic search for relevant peer-reviewed journal articles was conducted in 2022 using eight EBSCOhost databases. Fifty-two studies were included after screening was completed on 19,309 articles. Ageism-reduction interventions that assessed self-directed ageism outcomes were primarily implemented among college students (k = 20), older adults (k = 18), and children (k = 8). Despite methodological variation among included studies, such as measured domains of self-directed ageism, measures used, duration, dosage, and specific intervention characteristics, results suggest that purposeful in person intergenerational contact across age groups and in person intergenerational contact with aging education among college students and children are commonly effective in reducing self-directed ageism. Other interventions were found to be effective for certain age groups. Results provide initial support to suggest that intergenerational contact and aging education interventions can be similarly effective at reducing self-directed ageism as they are at reducing other-directed ageism. This scoping review also highlights several gaps in the literature. Notably, a universal definition for self-directed ageism is needed, a lifespan perspective of self-directed ageism should be taken, and research is needed to identify mechanisms of change responsible for significant outcomes achieved, especially within multifaceted interventions.

